# Measuring resilience to financial instability: A new dataset

**DOI:** 10.1016/j.dib.2016.11.012

**Published:** 2016-11-09

**Authors:** Domenico Lombardi, Pierre Siklos

**Affiliations:** Centre for International Governance Innovation and Wilfrid Laurier University, Canada

## Abstract

In recognition of the severe consequences of the recent international financial crisis, the topic of macroprudential policy has elicited considerable research effort. The data set reports, for 46 economies around the globe, an index of the capacity to deploy macroprudential policies. The index aims to represent the essence of what constitutes a macroprudential regime is developed and used in http://dx.doi.org/10.1016/j.jfs.2016.08.007 (D. Lombardi, P.L. Siklos, 2016) [1]. Specifically, the index quantifies: (1) how existing macroprudential frameworks are organized; and (2) how far a particular jurisdiction is from reaching the goals established by the Group of Twenty (G20) and the Financial Stability Board (FSB). The latter is a benchmark that has not been considered in the burgeoning literature that seeks to quantify the role of macroprudential policies.

**Specification Table**

**Table:** 

Subject area	*Economics*
More specific subject area	*Macroeconomics*
Type of data	*Excel spreadsheet*
How data was acquired	*Collected from original sources as described in*[1]
Data format	*Raw, filtered, and analyzed*
Experimental factors	*Collected from public sources*
Experimental features	*Characteristics of financial policies*
Data source location	*n/a*
Data accessibility	*Data is within this article*

**Value of the data**

• Allows replicability of key results in http://dx.doi.org/10.1016/j.jfs.2016.08.007.
• Points scholars in the direction of key sources of macroprudential policies.
• Promote additional data collection and extensions.

## 1 Data

The data were collected between mid-2014 and early 2015, primarily from central banks and the Financial Stability Board. The data quantify key features that constitute a macroprudential strategy intended to prevent financial instability. The data represent a mixture of quantitative and qualitative data at the source. For example, the number of macroprudential instruments is quantitative information as is the distance in time from FSB/G20 recommendations; authority to implement macroprudential policy is a dummy variable that is based on written information about the central bank׳s authority to implement such policies.

## 2 Experimental design, materials and methods

The data were created by assigning numerical codes to characteristics described in http://dx.doi.org/10.1016/j.jfs.2016.08.007 [1]. These characteristics are aggregated into an index that described macroprudential policies for 49 economies around the globe.
