# Supplementary material for: Measuring resilience to financial instability: A new dataset
Source: Data Brief. 2016 Nov 9;9:976–7. doi: 10.1016/j.dib.2016.11.012 (PMC5121165; doi:10.1016/j.dib.2016.11.012)
Supplement: Supplementary file 1 — Supplementary material [file mmc2.zip › MacroPru PAPER_AUGUST_2016_Appendix.docx]

**Online Appendix**

**Correlations between Lim et al. indexes, Cerutti MPI and the ALL, ALL + 2011 org changes (Table 2a)**

| Correlations | Lim et al. MaPP | p | Lim et al. MiPP | p | Lim et al. MoF | p | Cerutti MPI | p |
| --- | --- | --- | --- | --- | --- | --- | --- | --- |
| All (No dept.) Normalized | **0.007** | 0.973 | **0.139** | 0.480 | **0.003** | 0.987 | **0.008** | 0.958 |
| All (2011 dept.) Normalized | **0.069** | 0.726 | **0.123** | 0.534 | **0.032** | 0.872 | **0.041** | 0.801 |
| 7_median no dept. | **0.017** | 0.932 | **0.131** | 0.506 | **0.061** | 0.757 | **0.063** | 0.697 |
| 7_median 2011 dept. | **0.003** | 0.990 | **0.135** | 0.493 | **0.009** | 0.966 | **0.109** | 0.503 |
|  |  |  |  |  |  |  |  |  |
|  |  |  |  |  |  |  |  |  |
| Correlations | Lim et al. MaPP | p | Lim et al. MiPP | p | Lim et al. MoF | p |  |  |
| All (No dept.) Normalized | **0.007** | 0.973 | **0.139** | 0.480 | **0.003** | 0.987 |  |  |
| All (2011 dept.) Normalized | **0.069** | 0.726 | **0.123** | 0.534 | **0.032** | 0.872 |  |  |
| 7_median no dept. | **0.017** | 0.932 | **0.131** | 0.506 | **0.061** | 0.757 |  |  |
| 7_median 2011 dept. | **0.003** | 0.990 | **0.135** | 0.493 | **0.009** | 0.966 |  |  |
|  |  |  |  |  |  |  |  |  |
|  |  |  |  |  |  |  |  |  |
| Correlations | Cerutti MPI | p |  |  |  |  |  |  |
| All (No dept.) Normalized | **0.008** | 0.958 |  |  |  |  |  |  |
| All (2011 dept.) Normalized | **0.041** | 0.801 |  |  |  |  |  |  |
| 7_median no dept. | **0.063** | 0.697 |  |  |  |  |  |  |
| 7_median 2011 dept. | **0.109** | 0.503 |  |  |  |  |  |  |

7_ refers to all seven variants of the index (unweighted and weighted indexes). See text and Table 3.

**Table 2b. Index Values Under Different Assumptions About the Role of the Central Bank**

|  | ALL (No department details) | | FSB only (No department details) | |
| --- | --- | --- | --- | --- |
|  |  |  |  |  |
|  | MaP with  Decentralized Authority | MaP with  Centralized  Authority | MaP with  Decentralized Authority | MaP with  Centralized  Authority |
| Argentina | 12.50 | 5.50 | 9.61 | 2.61 |
| Australia | 14.67 | 2.67 | 11.29 | -0.71 |
| Austria | 13.50 | 1.50 | NA | NA |
| Belgium | 17.83 | 11.83 | NA | NA |
| Brazil | 14.25 | 4.25 | 12.14 | 2.14 |
| Canada | 13.83 | 1.83 | 11.48 | -0.52 |
| Chile | 12.50 | 1.50 | NA | NA |
| China | 21.33 | 4.33 | 17.45 | 0.45 |
| Colombia | 9.50 | -0.50 | NA | NA |
| Czech Republic | 18.42 | 16.42 | NA | NA |
| Denmark | 13.50 | -0.50 | NA | NA |
| Estonia | 17.00 | 9.00 | NA | NA |
| Finland | 9.00 | 1.00 | NA | NA |
| France | 14.50 | -3.50 | 13.00 | -5.00 |
| Germany | 12.92 | 3.92 | 12.77 | 3.77 |
| Hungary | 16.00 | 8.00 | NA | NA |
| Iceland | 12.00 | 3.00 | NA | NA |
| India | 18.67 | 0.67 | 15.75 | -2.25 |
| Indonesia | 20.92 | 10.92 | 17.23 | 7.23 |
| Ireland | 21.42 | 19.42 | NA | NA |
| Israel | 10.00 | 2.00 | NA | NA |
| Italy | 10.25 | 1.25 | 8.78 | -0.22 |
| Japan | 13.08 | 7.08 | 11.01 | 5.01 |
| Korea | 12.67 | 2.67 | 12.40 | 2.40 |
| Malaysia | 14.50 | 12.50 | NA | NA |
| Mexico | 12.08 | -1.92 | 8.77 | -5.23 |
| Netherlands | 21.00 | 14.00 | NA | NA |
| New Zealand | 13.75 | 11.75 | NA | NA |
| Norway | 10.00 | 3.00 | NA | NA |
| Peru | 2.00 | 0.00 | NA | NA |
| Poland | 10.00 | 4.00 | NA | NA |
| Portugal | 20.08 | 12.08 | NA | NA |
| Russia | 20.00 | 4.00 | 18.05 | 2.05 |
| Saudi Arabia | 6.50 | 5.50 | 2.62 | 1.62 |
| Singapore | 9.50 | 7.50 | 6.61 | 4.61 |
| Slovakia | 15.00 | 13.00 | NA | NA |
| Slovenia | 17.75 | 9.75 | NA | NA |
| South Africa | 13.50 | 5.50 | 8.62 | 0.62 |
| Spain | 12.50 | 2.50 | NA | NA |
| Sweden | 13.33 | 3.33 | NA | NA |
| Switzerland | 8.50 | 2.50 | NA | NA |
| Thailand | 14.17 | 12.17 | NA | NA |
| Turkey | 12.25 | 0.25 | 10.53 | -1.47 |
| UK | 18.08 | 12.08 | 17.33 | 11.33 |
| USA | 21.25 | -5.75 | 21.33 | -5.67 |
| EZ | 20.83 | 0.83 | 18.71 | -1.29 |

Note: index values (see Table 2a) depending on whether the index favours decentralization or centralization of authority in carrying out macroprudential policy.

**All Tables 3 below give the raw scores.**

**Table 3a. Implementing Macroprudential Policy: The Constituents of Financial System Stability**

|  | # of macroprudential instruments possessed and declared by CB | Is the CB free to implement available macroprudential instruments? | Is there government involvement in macroprudential instrument-related decisions? | Is macroprudential Implementation specified in primary, secondary legislation, or regulation? |
| --- | --- | --- | --- | --- |
| ‘Yes’ |  | 1 | 0 |  |
| ‘No’ |  | 0 | 1 |  |
| Other | # |  |  | Primary=1, Secondary=0.5, Regulation = 0.25, Other=0 |
| Argentina | 2.00 | 1.00 | 1.00 | 1.00 |
| Australia | 0.00 | 0.00 | 1.00 | 0.00 |
| Austria | 0.00 | 0.00 | 0.00 | 1.00 |
| Belgium | 6.00 | 1.00 | 0.00 | Draft in discussion - NA |
| Brazil | 4.00 | 1.00 | 0.00 | 0.25 |
| Canada | 0.00 | 0.00 | 0.00 | 0.25 |
| Chile | 0.00 | 0.00 | 0.00 | NS |
| China | 5.00 | 1.00 | 0.00 | 0.25 |
| Colombia | NS | NS | 0.00 | NS |
| Czech Republic | 6.00 | 1.00 | 0.00 | 1.00 |
| Denmark | 0.00 | 0.00 | 0.00 | 1.00 |
| Estonia | 3.00 | 1.00 | 0.00 | Draft in discussion - NA |
| Finland | 0.00 | 0.00 | 0.00 | 1.00 |
| France | 0.00 | 0.00 | 0.00 | 1.00 |
| Germany | 0.00 | 0.00 | 0.00 | 1.00 |
| Hungary | 0.00 | 0.00 | 0.00 | 1.00 |
| Iceland | 0.00 | 0.00 | 0.00 | NS |
| India | 4.00 | 0.00 | 0.00 | 0.25 |
| Indonesia | 6.00 | 1.00 | 0.00 | 0.25 |
| Ireland | 8.00 | 1.00 | 1.00 | 1.00 |
| Israel | 0.00 | 0.00 | 0.00 | NS |
| Italy | 0.00 | 0.00 | 0.00 | 1.00 |
| Japan | 0.00 | 0.00 | 0.00 | 1.00 |
| Korea | 0.00 | 0.00 | 0.00 | 0.25 |
| Malaysia | 4.00 | 1.00 | 0.00 | NS |
| Mexico | 0.00 | 0.00 | 0.00 | 0.50 |
| Netherlands | 8.00 | 1.00 | 0.00 | 0.50 |
| New Zealand | 4.00 | 1.00 | 1.00 | 1.00 |
| Norway | 0.00 | 0.00 | 0.00 | 1.00 |
| Peru | 0.00 | 0.00 | 0.00 | NS |
| Poland | 0.00 | 0.00 | 0.00 | 0.50 |
| Portugal | 8.00 | 1.00 | 0.00 | 1.00 |
| Russia | 5.00 | 1.00 | 0.00 | 1.00 |
| Saudi Arabia | 2.00 | 1.00 | 1.00 | NS |
| Singapore | 2.00 | 1.00 | 0.00 | 0.50 |
| Slovakia | 5.00 | 1.00 | 0.00 | 1.00 |
| Slovenia | 3.00 | 1.00 | 0.00 | 1.00 |
| South Africa | 0.00 | 1.00 | 0.00 | 0.50 |
| Spain | 0.00 | 0.00 | 0.00 | 0.50 |
| Sweden | 0.00 | 0.00 | 0.00 | 1.00 |
| Switzerland | 0.00 | 0.00 | 0.00 | NS |
| Thailand | 4.00 | 1.00 | 1.00 | NS |
| Turkey | 0.00 | 0.00 | 0.00 | 0.50 |
| UK | 3.00 | 1.00 | 0.00 | 1.00 |
| USA | 0.00 | 0.00 | 0.00 | 1.00 |
| EZ | 0.00 | 0.00 | 0.00 | 0.25 |

Note: “NS” indicates “not specified”, NA not applicable.

**Table 3bi. Coordination of Relevant Entities & Responsibility for Macroprudential Policy: Central Bank vs. Relevant Entities (Part 1) – Favours Decentralized Structure**

|  | Is CB the explicit macroprudential authority? | Is macroprudential policy explicitly in CB legislation? | Is there a financial stability/macroprudential coordinating body? | # (voting) members | # non-voting members | Is the CB a member? | Does CB chair coordination body? | If no body - CB share financial stability/macroprudentialmandate? |
| --- | --- | --- | --- | --- | --- | --- | --- | --- |
| ‘Yes’ | 1 | 1 | 1 |  |  | 1 | 1 | 1 |
| ‘No’ | 0 | 0 | 0 |  |  | 0 | 0 | 0 |
| Other |  |  |  | # | # / 2 |  |  |  |
| Argentina | NS | NS | 0.00 | NA | NA | NA | NA | 1.00 |
| Australia | 0.00 | 0.00 | 1.00 | 4.00 | NS | 1.00 | 1.00 | NA |
| Austria | 0.00 | 0.00 | 1.00 | 4.00 | NS | 1.00 | 0.00 | NA |
| Belgium | 1.00 | 0.50 | 0.00 | NA | NA | NA | NA | 1.00 |
| Brazil | 0.00 | 0.00 | 1.00 | 4.00 | NS | 1.00 | 0.00 | NA |
| Canada | 0.00 | 0.00 | 1.00 | 5.00 | NS | 1.00 | 0.00 | NA |
| Chile | 0.00 | 0.00 | 1.00 | 4.00 | 0.50 | 0.00 | 0.00 | NA |
| China | NS | 0.00 | 1.00 | 6.00 | 1.50 | 1.00 | 0.00 | NA |
| Colombia | NS | 0.00 | 1.00 | 4.00 | NS | 1.00 | 0.00 | NA |
| Czech Republic | 1.00 | 1.00 | 0.00 | NA | NA | NA | NA | 0.00 |
| Denmark | 0.00 | 0.00 | 1.00 | 5.00 | NS | 1.00 | 0.00 | NA |
| Estonia | 1.00 | 1.00 | 1.00 | 3.00 | NS | 1.00 | 0.00 | NA |
| Finland | 0.00 | 0.00 | 0.00 | NA | NA | NA | NA | 1.00 |
| France | 0.00 | 0.00 | 1.00 | 8.00 | NS | 1.00 | 0.00 | NA |
| Germany | 0.00 | 0.00 | 1.00 | 3.00 | 0.50 | 1.00 | 0.00 | NA |
| Hungary | 1.00 | 1.00 | 1.00 | 3.00 | NS | 1.00 | 0.50 | NA |
| Iceland | NS | 0.00 | 0.00 | NA | NA | NA | NA | 1.00 |
| India | NS | 0.00 | 1.00 | 8.00 | NS | 1.00 | 0.00 | NA |
| Indonesia | 1.00 | 0.00 | 1.00 | 4.00 | NS | 1.00 | 0.00 | NA |
| Ireland | 1.00 | 0.00 | 0.00 | NA | NA | NA | NA | 0.00 |
| Israel | 0.00 | 0.00 | 1.00 | 3.00 | NS | 1.00 | 0.00 | NA |
| Italy | 0.00 | 0.00 | 0.00 | NA | NA | NA | NA | 1.00 |
| Japan | NS | 0.00 | 1.00 | 2.00 | NS | 1.00 | NS | NA |
| Korea | 0.00 | 0.00 | 1.00 | 4.00 | NS | 1.00 | 0.00 | NA |
| Malaysia | 1.00 | 0.00 | 1.00 | 2.00 | NS | 1.00 | NS | NA |
| Mexico | 0.00 | 0.00 | 1.00 | 6.00 | NS | 1.00 | 0.00 | NA |
| Netherlands | 0.00 | 0.00 | 1.00 | 2.00 | 0.50 | 1.00 | 1.00 | NA |
| New Zealand | 1.00 | 0.00 | 0.00 | NA | NA | NA | NA | 0.00 |
| Norway | 0.00 | 0.00 | 0.00 | NA | NA | NA | NA | 1.00 |
| Peru | NS | 0.00 | 0.00 | NA | NA | NA | NA | 0.00 |
| Poland | 0.00 | 0.00 | 1.00 | 2.00 | NS | 1.00 | 0.00 | NA |
| Portugal | 1.00 | 1.00 | 0.00 | NA | NA | NA | NA | 1.00 |
| Russia | NS | 0.00 | 1.00 | 7.00 | NS | 1.00 | 0.00 | NA |
| Saudi Arabia | 1.00 | 0.00 | 0.00 | NA | NA | NA | NA | 0.00 |
| Singapore | NS | NS | 0.00 | NA | NA | NA | NA | 0.00 |
| Slovakia | 1.00 | 0.00 | 0.00 | NA | NA | NA | NA | 0.00 |
| Slovenia | 0.00 | 0.00 | 1.00 | 2.00 | 1.00 | 1.00 | 1.00 | NA |
| South Africa | NS | 0.00 | 1.00 | 3.00 | NS | 1.00 | 1.00 | NA |
| Spain | 0.00 | 0.00 | 1.00 | 4.00 | NS | 1.00 | 0.00 | NA |
| Sweden | 0.00 | 0.00 | 1.00 | 4.00 | NS | 1.00 | 0.00 | NA |
| Switzerland | NS | 0.00 | 0.00 | NA | NA | NA | NA | 1.00 |
| Thailand | 1.00 | 0.00 | 0.00 | NA | NA | NA | NA | 0.00 |
| Turkey | NS | 0.00 | 1.00 | 5.00 | NS | 1.00 | 0.00 | NA |
| UK | 1.00 | 1.00 | 0.00 | NA | NA | NA | NA | 1.00 |
| USA | 0.00 | 0.00 | 1.00 | 10.00 | 2.50 | 1.00 | 0.00 | NA |
| EZ | 0.00 | 0.00 | 1.00 | 8.00 | 1.00 | 1.00 | 1.00 | NA |

**Table 3bii. Coordination of Relevant Entities & Responsibility for Macroprudential Policy: Central Bank vs. Relevant Entities (Part 2) - Favours Decentralized Structure**

|  | If no body - CB sole owner of the mandate? | If no body - implicit coordination through MoUs? | If no body - …how many in total share? (incl. cb) | Is financial stability explicitly the responsibility of the CB? | …explicit in CB legislation? | Does the CB have an explicit definition of Fin Stab? | YES? In the legislation? | Elsewhere? |
| --- | --- | --- | --- | --- | --- | --- | --- | --- |
| ‘Yes’ | 0 | 0.5 |  | 1 | 1 | 1 | 1 | 0.5 |
| ‘No’ | 1 | 0 |  | 0 | 0 | 0 | 0 | 0 |
| Other |  |  | # / 2 |  | Indirect cases: 0.5 |  |  |  |
| Argentina | 1.00 | 0.00 | 1.50 | 1.00 | 1.00 | 0.00 | NA | NA |
| Australia | 1.00 | 0.00 | NA | 1.00 | 1.00 | 1.00 | 0.00 | 0.50 |
| Austria | 1.00 | 0.00 | NA | 1.00 | 1.00 | 1.00 | 0.00 | 0.50 |
| Belgium | 1.00 | 0.00 | 1.00 | 0.00 | 0.00 | 1.00 | 1.00 | 0.50 |
| Brazil | NA | NA | NA | 1.00 | 0.00 | 0.00 | NA | NA |
| Canada | NA | NA | NA | 1.00 | 1.00 | 1.00 | 0.00 | 0.50 |
| Chile | NA | NA | NA | 1.00 | 1.00 | 1.00 | 1.00 | 1.00 |
| China | NA | NA | NA | 1.00 | 1.00 | 0.00 | NA | NA |
| Colombia | NA | NA | NA | 1.00 | 0.00 | 1.00 | 0.00 | 0.50 |
| Czech Republic | 0.00 | 0.50 | 0.50 | 1.00 | 1.00 | 1.00 | 0.00 | 0.50 |
| Denmark | 1.00 | 0.00 | NA | 1.00 | 0.00 | 1.00 | 0.00 | 0.50 |
| Estonia | NA | NA | NA | 1.00 | 1.00 | 1.00 | 0.00 | 0.50 |
| Finland | 1.00 | 0.00 | 2.00 | 1.00 | 1.00 | 0.00 | NA | NA |
| France | NA | NA | NA | 1.00 | 1.00 | 0.00 | NA | NA |
| Germany | NA | NA | NA | 1.00 | 0.00 | 1.00 | 0.00 | 0.50 |
| Hungary | NA | NA | NA | 1.00 | 1.00 | 1.00 | 0.00 | 0.50 |
| Iceland | 1.00 | 0.00 | 2.50 | 1.00 | 1.00 | 1.00 | 0.00 | 0.50 |
| India | NA | NA | NA | 1.00 | 0.00 | 0.00 | 0.00 | NA |
| Indonesia | NA | NA | NA | 1.00 | NA | 1.00 | 0.00 | 0.50 |
| Ireland | 0.00 | 0.50 | 0.50 | 1.00 | 1.00 | 1.00 | 1.00 | 0.50 |
| Israel | NA | NA | NA | 1.00 | 1.00 | 0.00 | 0.00 | 0.00 |
| Italy | 1.00 | 0.50 | 2.00 | 1.00 | 1.00 | 0.00 | 0.00 | 0.00 |
| Japan | NA | NA | NA | 1.00 | 1.00 | 1.00 | 0.00 | 0.50 |
| Korea | NA | NA | NA | 1.00 | 0.00 | 1.00 | 0.00 | 0.50 |
| Malaysia | NA | NA | NA | 1.00 | 1.00 | 1.00 | 1.00 | 0.50 |
| Mexico | NA | NA | NA | 1.00 | 1.00 | 0.00 | NA | NA |
| Netherlands | NA | NA | NA | 1.00 | 1.00 | 1.00 | 0.00 | 0.50 |
| New Zealand | 0.00 | 0.50 | 0.50 | 1.00 | 1.00 | 0.00 | NA | NA |
| Norway | 1.00 | 0.00 | 1.50 | 1.00 | 0.00 | 1.00 | 0.00 | 0.50 |
| Peru | 1.00 | 0.00 | 0.00 | 0.00 | 0.00 | 0.00 | NA | NA |
| Poland | NA | NA | NA | 1.00 | 1.00 | 1.00 | 0.00 | 0.50 |
| Portugal | 1.00 | 0.00 | 2.00 | 1.00 | 1.00 | 0.00 | NA | NA |
| Russia | NA | NA | NA | 1.00 | 1.00 | 0.00 | NA | NA |
| Saudi Arabia | 0.00 | 0.00 | 0.50 | 1.00 | 0.00 | 0.00 | NA | NA |
| Singapore | 0.00 | 0.50 | 0.50 | 1.00 | 1.00 | 0.00 | 0.00 | 0.00 |
| Slovakia | 0.00 | 0.50 | 0.50 | 1.00 | 1.00 | 1.00 | 0.00 | 0.50 |
| Slovenia | NA | NA | NA | 1.00 | 1.00 | 1.00 | 0.00 | 0.50 |
| South Africa | NA | NA | NA | 1.00 | 0.50 | 1.00 | 0.00 | 0.50 |
| Spain | NA | NA | NA | 1.00 | 1.00 | 1.00 | 0.00 | 0.50 |
| Sweden | NA | NA | NA | 1.00 | 0.50 | 1.00 | 0.00 | 0.50 |
| Switzerland | 1.00 | 0.00 | 1.00 | 1.00 | 1.00 | 1.00 | 0.00 | 0.50 |
| Thailand | 0.00 | 0.50 | 0.50 | 1.00 | 1.00 | 0.00 | 0.00 | 0.00 |
| Turkey | NA | NA | NA | 1.00 | 1.00 | 0.00 | NA | NA |
| UK | 1.00 | 0.00 | 1.00 | 1.00 | 1.00 | 0.00 | NA | NA |
| USA | NA | NA | NA | 1.00 | 1.00 | 0.00 | NA | NA |
| EZ | NA | NA | NA | 1.00 | 0.00 | 1.00 | 0.00 | 0.50 |

**Table 3c. Deposit Insurance**

|  | Is there Deposit Insurance? |
| --- | --- |
| ‘Yes’ | 1 |
| ‘No’ | 0 |
| Argentina | 1.00 |
| Australia | 1.00 |
| Austria | 1.00 |
| Belgium | 1.00 |
| Brazil | 1.00 |
| Canada | 1.00 |
| Chile | 1.00 |
| China | 1.00 |
| Colombia | 1.00 |
| Czech Republic | 1.00 |
| Denmark | 1.00 |
| Estonia | 1.00 |
| Finland | 1.00 |
| France | 1.00 |
| Germany | 1.00 |
| Hungary | 1.00 |
| Iceland | 1.00 |
| India | 1.00 |
| Indonesia | 1.00 |
| Ireland | 1.00 |
| Israel | 1.00 |
| Italy | 1.00 |
| Japan | 1.00 |
| Korea | 1.00 |
| Malaysia | 1.00 |
| Mexico | 1.00 |
| Netherlands | 1.00 |
| New Zealand | 0.00 |
| Norway | 1.00 |
| Peru | 1.00 |
| Poland | 1.00 |
| Portugal | 1.00 |
| Russia | 1.00 |
| Saudi Arabia | 0.00 |
| Singapore | 1.00 |
| Slovakia | 1.00 |
| Slovenia | 1.00 |
| South Africa | 1.00 |
| Spain | 1.00 |
| Sweden | 1.00 |
| Switzerland | 1.00 |
| Thailand | 1.00 |
| Turkey | 1.00 |
| UK | 1.00 |
| USA | 1.00 |
| EZ | 1.00 |

**Table 3d. Transparency & Accountability: Speeches, Media Releases, Financial Stability Reports**

|  | Going back to 2008, # of speeches with 'macroprudential' in title | Going back to 2008, # of press releases with 'macroprudential' in title | Does the Central Bank publish a separate Financial Stability Report? | …is there a section explicitly devoted to macroprudential policy? | Does an authority other than the Central Bank publish a separate Financial Stability Report? | …is there a section explicitly devoted to macroprudential policy? |
| --- | --- | --- | --- | --- | --- | --- |
| ‘Yes’ |  |  | 1 | 0.5 | 0.5 | 0.25 |
| ‘No’ |  |  | 0 | 0 | 0 | 0 |
| ‘Other’ | # / 12 | # / 12 |  |  |  |  |
| Argentina | 0.00 | 0.00 | 1.00 | 0.00 | 0.00 | NA |
| Australia | 0.17 | 0.00 | 1.00 | 0.00 | 0.00 | NA |
| Austria | 0.00 | 0.00 | 1.00 | 0.00 | 0.00 | NA |
| Belgium | 0.00 | 0.33 | 1.00 | 0.50 | 0.00 | NO |
| Brazil | 0.00 | 0.00 | 0.00 | 0.00 | 0.00 | NA |
| Canada | 0.00 | 0.08 | 1.00 | 0.00 | 0.00 | NA |
| Chile | 0.00 | 0.00 | 1.00 | 0.00 | 0.00 | NA |
| China | 0.08 | 0.00 | 1.00 | 0.50 | 0.00 | NA |
| Colombia | 0.00 | 0.00 | 0.00 | 0.00 | 0.00 | NA |
| Czech Republic | 0.42 | 0.00 | 1.00 | 0.50 | 0.00 | NA |
| Denmark | 0.00 | 0.00 | 1.00 | 0.00 | 0.00 | NA |
| Estonia | 0.00 | 0.00 | 1.00 | 0.00 | 0.50 | 0.00 |
| Finland | 0.00 | 0.00 | 1.00 | 0.00 | 0.00 | NA |
| France | 0.50 | 0.00 | 0.00 | NA | 0.00 | NA |
| Germany | 0.50 | 0.17 | 1.00 | 0.50 | 0.50 | 0.25 |
| Hungary | 0.00 | 0.00 | 1.00 | 0.50 | 0.50 | 0.00 |
| Iceland | 0.00 | 0.00 | 1.00 | 0.50 | 0.50 | 0.00 |
| India | 0.83 | 0.08 | 1.00 | 0.00 | 0.50 | 0.00 |
| Indonesia | 0.08 | 0.08 | 1.00 | 0.50 | 0.50 | 0.00 |
| Ireland | 0.08 | 0.33 | 0.00 | 0.50 | 0.00 | NA |
| Israel | 0.00 | 0.00 | 1.00 | 0.00 | 0.00 | NA |
| Italy | 0.25 | 0.00 | 1.00 | 0.00 | 0.50 | 0.00 |
| Japan | 0.58 | 0.00 | 1.00 | 0.50 | 0.50 | 0.00 |
| Korea | 0.42 | 0.25 | 1.00 | 0.50 | 0.50 | 0.25 |
| Malaysia | 0.00 | 0.00 | 1.00 | 0.00 | 0.00 | NA |
| Mexico | 0.08 | 0.00 | 0.00 | 0.00 | 0.50 | 0.00 |
| Netherlands | 0.00 | 0.00 | 1.00 | 0.50 | 0.00 | NA |
| New Zealand | 0.33 | 0.42 | 1.00 | 0.00 | 0.00 | NA |
| Norway | 0.50 | 0.00 | 1.00 | 0.00 | 0.50 | 0.00 |
| Peru | 0.00 | 0.00 | 0.00 | NA | 0.00 | NA |
| Poland | 0.00 | 0.00 | 1.00 | 0.00 | 0.00 | NA |
| Portugal | 0.00 | 0.08 | 1.00 | 0.00 | 0.00 | NA |
| Russia | 0.00 | 0.00 | 1.00 | 0.00 | 0.00 | NA |
| Saudi Arabia | 0.00 | 0.00 | 0.00 | NA | 0.00 | NA |
| Singapore | 0.00 | 0.00 | 1.00 | 0.00 | 0.00 | NA |
| Slovakia | 0.00 | 0.00 | 1.00 | 0.50 | 0.00 | NA |
| Slovenia | 0.00 | 0.00 | 1.00 | 0.50 | 0.50 | 0.25 |
| South Africa | 0.00 | 0.00 | 1.00 | 0.00 | 0.00 | NA |
| Spain | 0.00 | 0.00 | 1.00 | 0.50 | 0.00 | NA |
| Sweden | 0.33 | 0.00 | 1.00 | 0.00 | 0.00 | NA |
| Switzerland | 0.00 | 0.00 | 1.00 | 0.00 | 0.00 | NA |
| Thailand | 0.08 | 0.08 | 1.00 | 0.00 | 0.00 | NA |
| Turkey | 0.17 | 0.08 | 1.00 | 0.50 | 0.00 | NA |
| UK | 1.17 | 0.42 | 1.00 | 0.50 | 0.00 | NA |
| USA | 0.25 | 0.00 | 0.00 | NA | 0.50 | 0.00 |
| EZ | 1.67 | 0.17 | 1.00 | 0.50 | 0.50 | 0.25 |

**Table 3e. Governance of Macroprudential Policies**

|  | Number of Monetary Policy Committee members (if applicable) | Number of Financial Stability Committee members (if applicable) | Is there a separate Financial Stability Committee? | Change in # of "major" departments based on the available organizational charts since 2001* | Change in # of "major" departments based on the available organizational charts since 2011** | Is there a distinct financial stability department? | If so, is it on the same 'level' as department responsible for MP? | Does it report to the same deputy governor? |
| --- | --- | --- | --- | --- | --- | --- | --- | --- |
| ‘Yes’ |  |  | 1 |  |  | 1 | 1 | 1 |
| ‘No’ |  |  | 0 |  |  | 0 | 0 | 0 |
| ‘Other’ | # | # |  | # | # |  |  |  |
| Argentina | 5.00 | NA | 0.00 | NA | 3.00 | 0.00 | NA | NA |
| Australia | 9.00 | NA | 0.00 | 0.00 | 1.00 | 1.00 | 1.00 | 0.00 |
| Austria | 24.00 | NA | 0.00 | 0.00 | 1.00 | 1.00 | 0.00 | NA |
| Belgium | 24.00 | NA | 0.00 | 1.00 | 2.00 | 1.00 | NA | NA |
| Brazil | 9.00 | 8.00 | 1.00 | 12.00 | 1.00 | 1.00 | 1.00 | 0.00 |
| Canada | 6.00 | NA | 0.00 | 1.00 | 1.00 | 1.00 | 1.00 | 0.00 |
| Chile | 5.00 | NA | 0.00 | -6.00 | 0.00 | 1.00 | 1.00 | 0.00 |
| China | 13.00 | NA | 0.00 | NA | NA | 1.00 | 1.00 | 0.00 |
| Colombia | 7.00 | NA | 0.00 | NA | 1.00 | 1.00 | NS | NA |
| Czech Republic | 7.00 | NA | 0.00 | 1.00 | 0.00 | 1.00 | 1.00 | 0.00 |
| Denmark | 3.00 | NA | 0.00 | -4.00 | -2.00 | 1.00 | 1.00 | 0.00 |
| Estonia | 24.00 | NA | 0.00 | -8.00 | -3.00 | 1.00 | NS | NA |
| Finland | 24.00 | NA | 0.00 | 7.00 | 0.00 | 1.00 | 1.00 | 0.00 |
| France | 24.00 | NA | 0.00 | 1.00 | 0.00 | 1.00 | 1.00 | 0.00 |
| Germany | 24.00 | NA | 0.00 | 2.00 | 2.00 | 1.00 | 1.00 | 1.00 |
| Hungary | 9.00 | NA | 0.00 | 5.00 | 5.00 | 1.00 | 1.00 | 1.00 |
| Iceland | 5.00 | NA | 0.00 | NA | NA | 1.00 | 1.00 | 0.00 |
| India | 7.00 | NA | 0.00 | 8.00 | 4.00 | 1.00 | 1.00 | 0.00 |
| Indonesia | 7.00 | NA | 0.00 | NA | 0.00 | 1.00 | 1.00 | 0.00 |
| Ireland | 24.00 | 1.00 | 1.00 | -4.00 | 2.00 | 1.00 | 1.00 | 1.00 |
| Israel | 6.00 | NA | 0.00 | -5.00 | 0.00 | 0.00 | NA | NA |
| Italy | 24.00 | NA | 0.00 | -3.00 | -3.00 | 1.00 | 1.00 | 1.00 |
| Japan | 9.00 | NA | 0.00 | -1.00 | 0.00 | 1.00 | 1.00 | 0.00 |
| Korea | 7.00 | NA | 0.00 | 0.00 | -1.00 | NS | NS | NS |
| Malaysia | 10.00 | NA | 0.00 | 3.00 | 1.00 | 1.00 | 1.00 | 0.00 |
| Mexico | 5.00 | NA | 0.00 | 5.00 | 0.00 | 1.00 | 1.00 | 0.00 |
| Netherlands | 24.00 | NA | 0.00 | 2.00 | 0.00 | 1.00 | 1.00 | 0.00 |
| New Zealand | 1.00 | NA | 0.00 | 0.00 | 1.00 | 1.00 | 1.00 | 0.00 |
| Norway | 7.00 | NA | 0.00 | 6.00 | 2.00 | 1.00 | 1.00 | 1.00 |
| Peru | 7.00 | NA | 0.00 | 0.00 | 0.00 | 1.00 | 1.00 | 1.00 |
| Poland | 10.00 | NA | 0.00 | 5.00 | 0.00 | 1.00 | 0.00 | 0.00 |
| Portugal | 24.00 | NA | 0.00 | 4.00 | 2.00 | 1.00 | 1.00 | 0.00 |
| Russia | 15.00 | NA | 0.00 | 8.00 | 7.00 | 1.00 | 1.00 | 1.00 |
| Saudi Arabia | 5.00 | NA | 0.00 | 0.00 | 0.00 | 1.00 | 1.00 | 1.00 |
| Singapore | 11.00 | 1.00 | 1.00 | 0.00 | 0.00 | 1.00 | 0.00 | 0.00 |
| Slovakia | 24.00 | NA | 0.00 | -5.00 | 0.00 | 1.00 | 1.00 | 0.00 |
| Slovenia | 24.00 | NA | 0.00 | -1.00 | 0.00 | 1.00 | NS | NS |
| South Africa | 7.00 | 3.00 | 1.00 | NA | NA | NS | NS | NS |
| Spain | 24.00 | NA | 0.00 | -1.00 | 0.00 | 1.00 | 1.00 | 0.00 |
| Sweden | 6.00 | NA | 0.00 | -6.00 | 0.00 | 1.00 | 1.00 | 0.00 |
| Switzerland | 11.00 | NA | 0.00 | 14.00 | 9.00 | 0.00 | NA | NA |
| Thailand | 7.00 | 1.00 | 1.00 | 1.00 | -1.00 | 1.00 | 1.00 | 0.00 |
| Turkey | 7.00 | NA | 0.00 | NA | 0.00 | 1.00 | 1.00 | 0.00 |
| UK | 9.00 | 10.00 | 1.00 | NA | NA | 1.00 | 1.00 | 0.00 |
| USA | 10.00 | 3.00 | 1.00 | 3.00 | -1.00 | 1.00 | 1.00 | 0.00 |
| EZ | 24.00 | NA | 0.00 | 9.00 | 3.00 | 1.00 | 1.00 | 0.00 |

**Table 3f. The Place of Macroprudential Policy in the Monetary Transmission Mechanism**

|  | Does the CB draw a link/links between Monetary Policy and Macroprudential Policy? |
| --- | --- |
| ‘Yes’ | 1 |
| ‘No’ | 0 |
| Argentina | 0.00 |
| Australia | 0.00 |
| Austria | 0.00 |
| Belgium | 1.00 |
| Brazil | 0.00 |
| Canada | 1.00 |
| Chile | 0.00 |
| China | 1.00 |
| Colombia | 0.00 |
| Czech Republic | 1.00 |
| Denmark | 0.00 |
| Estonia | 0.00 |
| Finland | 0.00 |
| France | 0.00 |
| Germany | 0.00 |
| Hungary | 1.00 |
| Iceland | 1.00 |
| India | 0.00 |
| Indonesia | 1.00 |
| Ireland | 1.00 |
| Israel | 1.00 |
| Italy | 0.00 |
| Japan | 1.00 |
| Korea | 0.00 |
| Malaysia | 0.00 |
| Mexico | 0.00 |
| Netherlands | 0.00 |
| New Zealand | 1.00 |
| Norway | 0.00 |
| Peru | 0.00 |
| Poland | 0.00 |
| Portugal | 0.00 |
| Russia | 0.00 |
| Saudi Arabia | 0.00 |
| Singapore | 0.00 |
| Slovakia | 0.00 |
| Slovenia | 0.00 |
| South Africa | 0.00 |
| Spain | 0.00 |
| Sweden | 1.00 |
| Switzerland | 0.00 |
| Thailand | 1.00 |
| Turkey | 0.00 |
| UK | 1.00 |
| USA | 1.00 |
| EZ | 1.00 |

**Table 3g. Distance to FSB/G20 Macroprudential Recommendations**

|  | Regulatory Framework for Macroprudential Oversight | System-wide monitoring and the use of macroprudential instruments | Improved cooperation between supervisors and central banks |
| --- | --- | --- | --- |
|  | As per FSB Survey Responses | | |
|  | Primary=1, Secondary=.5, Regulation=.25, Other=0 | | |
| Argentina | 0.25 | 1.25 | 0.50 |
| Australia | 0.00 | 0.00 | 1.00 |
| Austria | NA | NA | NA |
| Belgium | NA | NA | NA |
| Brazil | 0.25 | 0.25 | 0.00 |
| Canada | 0.25 | 0.25 | 0.25 |
| Chile | NA | NA | NA |
| China | 0.00 | 0.25 | 0.50 |
| Colombia | NA | NA | NA |
| Czech Republic | NA | NA | NA |
| Denmark | NA | NA | NA |
| Estonia | NA | NA | NA |
| Finland | NA | NA | NA |
| France | 1.00 | 1.00 | 1.00 |
| Germany | 1.00 | 1.00 | 1.00 |
| Hungary | NA | NA | NA |
| Iceland | NA | NA | NA |
| India | 0.00 | 0.25 | 0.00 |
| Indonesia | 0.75 | 0.25 | 0.00 |
| Ireland | NA | NA | NA |
| Israel | NA | NA | NA |
| Italy | 1.00 | 1.00 | 0.00 |
| Japan | 0.00 | 1.00 | 0.00 |
| Korea | 0.25 | 0.25 | 1.00 |
| Malaysia | NA | NA | NA |
| Mexico | 1.00 | 0.50 | 0.50 |
| Netherlands | NA | NA | NA |
| New Zealand | NA | NA | NA |
| Norway | NA | NA | NA |
| Peru | NA | NA | NA |
| Poland | NA | NA | NA |
| Portugal | NA | NA | NA |
| Russia | 1.00 | 1.00 | 1.00 |
| Saudi Arabia | 0.00 | 0.00 | 0.00 |
| Singapore | 1.00 | 0.75 | 0.00 |
| Slovakia | NA | NA | NA |
| Slovenia | NA | NA | NA |
| South Africa | 0.25 | 0.75 | 0.25 |
| Spain | NA | NA | NA |
| Sweden | NA | NA | NA |
| Switzerland | NA | NA | NA |
| Thailand | NA | NA | NA |
| Turkey | 1.00 | 0.75 | 0.25 |
| UK | 1.00 | 1.00 | 1.25 |
| USA | 1.25 | 1.25 | 1.00 |
| EZ | 1.25 | 1.25 | 0.25 |

**Table 3h. Response Time to FSB Recommendations**

|  | Response Time |
| --- | --- |
|  | Timeliness |
| Argentina | 4.89 |
| Australia | 4.38 |
| Austria | NA |
| Belgium | NA |
| Brazil | 2.61 |
| Canada | 3.10 |
| Chile | NA |
| China | 4.63 |
| Colombia | NA |
| Czech Republic | NA |
| Denmark | NA |
| Estonia | NA |
| Finland | NA |
| France | 4.50 |
| Germany | 3.14 |
| Hungary | NA |
| Iceland | NA |
| India | 3.17 |
| Indonesia | 4.69 |
| Ireland | NA |
| Israel | NA |
| Italy | 3.47 |
| Japan | 3.08 |
| Korea | 1.76 |
| Malaysia | NA |
| Mexico | 5.32 |
| Netherlands | NA |
| New Zealand | NA |
| Norway | NA |
| Peru | NA |
| Poland | NA |
| Portugal | NA |
| Russia | 4.95 |
| Saudi Arabia | 3.88 |
| Singapore | 4.64 |
| Slovakia | NA |
| Slovenia | NA |
| South Africa | 6.13 |
| Spain | NA |
| Sweden | NA |
| Switzerland | NA |
| Thailand | NA |
| Turkey | 3.72 |
| UK | 4.00 |
| USA | 3.42 |
| EZ | 4.87 |

Correlations between Indexes

* represents significance at the 0.05 level

Relevant Codes: XXX_YYY_ZZZ

| Element | Possible Values | Description |
| --- | --- | --- |
| XXX | all, fsb | All countries, or FSB-members (FSB members have ‘Timeliness’ and ‘Distance’ features |
| YYY | na, 2001, 2011 | Changes in department details |
| ZZZ | 0, 1, 2, 3, 12, 13, 23, 123 | Sensitivity considerations, and combinations specifically:   - 0 Original Index - 1 Responsibility - 2 Removal of transmission mechanism feature - 3 Removal of # instruments feature - 12, 13, 23, 123 combinations of 1 2 and 3 |

**UNWEIGHTED**

**All Countries (No dept. details), unweighted, individual**

**All Countries (2001 dept. details), unweighted, individual**

**All Countries (2011 dept. details), unweighted, individual**

**FSB Countries (No dept. details), unweighted, individual**

**FSB Countries (2001 dept. details), unweighted, individual**

**FSB Countries (2011 dept. details), unweighted, individual**

**Correlations between Decentralized and Centralized Versions of the Index**

All Economies

| Sample: 1 46 | |  |  |
| --- | --- | --- | --- |
| Included observations: 46 | | |  |
|  |  |  |  |
|  |  |  |  |
| Correlation | |  |  |
| t-Statistic | |  |  |
| Probability | Decentralized | Centralized |  |
| Decentralized | 1.000000 |  |  |
|  | ----- |  |  |
|  | ----- |  |  |
|  |  |  |  |
| Centralized | 0.409722 | 1.000000 |  |
|  | 2.979345 | ----- |  |
|  | 0.0047 | ----- |  |
|  |  |  |  |
|  |  |  |  |

FSB Members only

| Sample: 1 46 | |  |
| --- | --- | --- |
| Included observations: 21 | | |
| Balanced sample (listwise missing value deletion) | | |
|  |  |  |
|  |  |  |
| Correlation | |  |
| t-Statistic | |  |
| Probability | Decentralized | Centralized |
| Decentralized | 1.000000 |  |
|  | ----- |  |
|  | ----- |  |
|  |  |  |
| Centralized | -0.011578 | 1.000000 |
|  | -0.050471 | ----- |
|  | 0.9603 | ----- |
|  |  |  |
|  |  |  |

**Financial Stability Definitions**

| **Country** | **Legislation** | **Policy Report** | **FSR Recent** | **FSR Older** | **Annual Report** | **Non-official doc (E.g., website)** |
| --- | --- | --- | --- | --- | --- | --- |
| Argentina |  |  |  |  |  |  |
| Australia | NO |  | NO |  | NO | http://www.rba.gov.au/fin-stability/about.html |
| Austria | NO |  | X |  |  | http://www.oenb.at/en/Financial-Stability.html |
| Belgium | X |  |  |  |  |  |
| Brazil | NO |  | NO | NO |  | NO |
| Canada |  |  | X JUNE 2013 |  |  |  |
| Chile |  |  | NO | NO |  |  |
| China | NO |  |  |  |  |  |
| Colombia |  |  |  | X SEP 2011 |  |  |
| Czech Republic |  |  | X 2013/2014 |  |  |  |
| Denmark |  |  | X 2014 |  |  |  |
| Estonia |  |  | NO |  |  | Website - http://www.eestipank.ee/en/eesti-panks-role-safeguarding-financial-stability |
| Finland |  |  | X 2012 |  |  | Website - http://www.suomenpankki.fi/en/rahoitusjarjestelman_vakaus/Pages/default.aspx |
| France |  |  | NO |  | NO |  |
| Germany |  |  | X 2014 |  |  |  |
| Hungary |  |  | X 2014 |  |  |  |
| Iceland |  |  | X 2014 |  |  |  |
| India | NO |  | NO |  |  |  |
| Indonesia |  |  | NO |  |  |  |
| Ireland |  |  |  |  |  |  |
| Israel |  |  |  |  |  |  |
| Italy | NO | NO | NO | NO | NO |  |
| Japan |  |  | NO |  |  |  |
| Korea |  |  |  |  |  |  |
| Malaysia | X |  |  |  |  |  |
| Mexico | NO | NO | NO | NO | NO |  |
| Netherlands |  |  |  |  |  |  |
| New Zealand |  |  |  |  |  |  |
| Norway |  |  | X 2014 |  |  |  |
| Peru | NO | NO | NO | NO | NO |  |
| Poland |  |  |  |  |  |  |
| Portugal | NO | NO | NO | NO | NO |  |
| Russia | NO | NO | NO | NO | NO |  |
| Saudi Arabia | NO | NO | NO | NO | NO |  |
| Singapore |  |  |  |  |  |  |
| Slovakia |  |  | X 2014 |  |  |  |
| Slovenia | NO |  | NO |  |  | Website - http://www.bsi.si/en/financial-stability.asp?MapaId=1189 |
| South Africa |  |  |  |  |  |  |
| Spain |  |  |  |  |  |  |
| Sweden |  |  | X |  |  |  |
| Switzerland |  |  |  | X 2010 |  |  |
| Thailand |  |  |  |  |  |  |
| Turkey |  |  | NO |  |  |  |
| UK |  |  | X 2014 (vague) |  |  |  |
| USA |  |  |  |  |  |  |
| EZ |  |  | X 2014 |  |  |  |

**Text of definitions found**

Austria

Financial stability means that the financial system – financial intermediaries, financial markets and financial infrastructures – is capable of ensuring the efficient allocation of financial resources and fulfilling its key macroeconomic functions even if financial imbalances and shocks occur. Under conditions of financial stability, economic agents have confidence in the banking system and have ready access to financial services, such as payments, lending, deposits and hedging.

Belgium (translation from CB Legislation)

"Stability of the financial system" means a situation where the probability discontinuity or disruption of the functioning of the financial system slight or, if such disturbances would occur, where the impact on the economy would be limited;

Canada

Financial stability is defined as the resilience of the financial system to unanticipated adverse shocks, which enables the continued smooth functioning of the financial intermediation process.

Colombia

Maintaining financial stability … is understood as a situation in which the financial system is able to broker financial flows effectively.

Czech Republic

The CNB defines financial stability as a situation where the financial system operates with no serious failures or undesirable impacts on the present and future development of the economy as a whole, while showing a high degree of resilience to shocks. The CNB’s definition is based on the fact that financial stability may be disturbed both by processes inside the financial sector that lead to the emergence of weak spots, and by strong shocks, which may arise from the external environment, domestic macroeconomic developments, large debtors and creditors, economic policies or changes in the institutional environment.

Denmark

Danmarks Nationalbank defines financial stability as a condition whereby the overall financial system is robust enough for any problems within the sector not to spread and prevent the financial system from functioning as an efficient provider of capital and financial services.

Finland

A stable financial system is capable of operating beyond reproach, of handling its basic tasks, such as the undisturbed transmission of finance and payments, pricing of financial instruments and efficient distribution of risk. Furthermore, the risk-bearing capacity of the financial market agents and public confidence in financial institutions and the financial markets must be sufficient to endure even larger disruptions in the operating environment.

Germany

The Bundesbank defines financial stability as the financial system’s ability to perform its key macroeconomic functions, especially in periods of stress and upheaval. The objective of financial stability policy is to strengthen the resilience of the financial system. Traditional banking supervision aims to ensure the stability of individual institutions. This means that risks to the economy as a whole arising from individual institutions or groups of institutions that are experiencing distress are not the main focus

Hungary

Financial stability is a state in which the financial system, including key financial markets and financial institutions, is capable of withstanding economic shocks and can fulfil its key functions smoothly, i.e. intermediating financial resources, managing financial risks and processing payment transactions

Iceland

Financial stability means that the financial system is equipped to withstand shocks to the economy and financial markets, to mediate credit and payments, and to redistribute risks appropriately.

Malaysia (CB Legislation)

“risk to financial stability” means a risk which in the opinion of the Bank disrupts, or is likely to disrupt, the financial intermediation process including the orderly functioning of the money market and foreign exchange market, or affects, or is likely to affect, public confidence in the financial system or the stability of the financial system.

Norway

Financial stability implies a financial system that is resilient to shocks and thus capable of channelling funds, executing payments and distributing risk efficiently.

Slovakia

The financial sector is deemed to be stable when it is able to smoothly fulfil its core functions, even amidst substantial adverse shocks in the external or domestic economic and financial environment. At the same time, financial sector stability is perceived as a necessary condition for sound functioning of the real economy.

Sweden

The Riksbank defines financial stability as meaning that the financial system is able to maintain its basic functions – the mediation of payments, the conversion of savings into funding and risk management – and is also resilient to disruptions that threaten these functions.

Switzerland

A stable financial system can be defined as a system which fulfils its functions and is able to withstand the shocks to which it is exposed.

UK

Financial stability objective relates primarily to the identification of, monitoring of, and taking of action to remove or reduce systemic risks with a view to protecting and enhancing the resilience of the UK financial system

EZ

A condition in which the financial system – intermediaries, markets and market infrastructures – can withstand shocks without major disruption in financial intermediation and in the effective allocation of savings to productive investment.
