# Supplementary material for: Measuring resilience to financial instability: A new dataset
Source: Data Brief. 2016 Nov 9;9:976–7. doi: 10.1016/j.dib.2016.11.012 (PMC5121165; doi:10.1016/j.dib.2016.11.012)
Supplement: Supplementary file 2 — Supplementary material [file mmc1.pdf]

“The authors declare that they have no relevant or material financial interests that relate to the research described in this paper”
